# 2D Virtual Reality-Based Exercise Improves Spatial Navigation in Institutionalized Non-robust Older Persons: A Preliminary Data Report of a Single-Blind, Randomized, and Controlled Study

**DOI:** 10.3389/fneur.2020.609988

**Published:** 2021-01-18

**Authors:** Luciana Mendes Oliveira, Eric Hudson Evangelista e Souza, Mariana Rocha Alves, Lara S. F. Carneiro, Daniel Ferreira Fagundes, Alfredo Maurício Batista de Paula, Knut Engedal, Osvaldo J. M. Nascimento, Renato Sobral Monteiro-Junior

**Affiliations:** ^1^Graduate Program of Medicine (Neurology/Neurosciences), Federal Fluminense University, Niterói, Brazil; ^2^Graduate Program of Health Sciences, Montes Claros State University, Montes Claros, Brazil; ^3^Universitary Institute of Maia, Maia, Portugal; ^4^Norwegian Advisory Unit for Ageing and Health, Vestfold Hospital Trust, Tønsberg, Norway

**Keywords:** dementia, spatial orientation, frailty, physical activity, video games

## Abstract

**Background:** Spatial navigation is a prodromal dementia marker. Exercise used alongside virtual reality improves many cognitive functions, but effects on spatial navigation are still unclear.

**Objective:** To investigate the effect of virtual reality-based physical exercise with 2D exergames on spatial navigation in institutionalized non-robust older persons.

**Method:** A total of 14 older persons (aged ≧ 60) were randomly allocated to the exergame (EG) and active control (ACG) groups. EG performed exercises with 2D exergames, while the ACG used the same movements as the EG, but without the use of virtual reality. Spatial navigation was assessed through the Floor Maze Test, where the immediate maze time (IMT) and delayed maze time (DMT) were recorded.

**Results:** Spatial navigation was enhanced in EG participants compared to ACG individuals. A significant (*p* = 0.01) IMT reduction between groups was observed, while DMT time without prior planning was significantly different at the significance threshold (*p* = 0.07).

**Conclusions:** Virtual reality-based exercise improves the spatial navigation of institutionalized non-robust older persons. This study should be replicated to confirm the findings reported herein.

**Clinical Trial Registration:** This study was registered in the Brazilian Registry of Clinical Trials (Protocol RBR-8dv3kg - https://ensaiosclinicos.gov.br/rg/RBR-8dv3kg).

## Introduction

The development of chronic diseases in older persons is common, harming their functional capacity and resulting in loss of autonomy (1). Independence reduction is the main reason why older persons dwell in long-term care institutions (LTCIs) (2).

In Brazil, over half of institutionalized older persons have dementia (3), which may occur after institutionalization (4). Spatial navigation is among the cognitive functions that deteriorate in dementia cases and compromises the older person's ability of locomotion (5), which in turn is associated with prodromal dementia (6). Spatial navigation is defined as the ability to integrate cognitive processes and sensorial systems, especially the visual system, into environment data processing and body positioning during spatial displacement (5).

An interesting method used to stimulate spatial navigation ability is immersive virtual reality-based navigation training (VR) (7), which involves the activation of brain zones associated with cognitive processes, such as the hippocampus, caudate nucleus, and frontal cortex (8). However, the use of VR immersive systems is complex and expensive, and difficult to apply in older persons living in LTCIs. Previous studies have indicated that active video games associating two-dimensional virtual environment digital games with physical exercise (2D exergames) improve short-term memory, executive functions, sensorimotor integration, and mobility in older individuals (9, 10). To date, however, no robust evidence that 2D exergames might alter spatial navigation in older individuals is available. In this context, this study aimed to analyze the effect of 2D exergame training on the spatial navigation in institutionalized non-robust older individuals.

## Methods

### Trial Design

This trial comprised a controlled pilot study with blind randomization of two parallel groups. Consolidated Standards of Reporting Trials (CONSORT) were met. The study protocol was registered in the Brazilian Registration of Clinical Trials (ReBEC), under alphanumeric code number RBR-8dv3kg (https://ensaiosclinicos.gov.br/rg/RBR-8dv3kg).

### Participants

The sample was comprised of older persons inhabiting four LTCIs in the Brazilian cities of Montes Claros/MG and Rio de Janeiro/RJ. Both men and women were recruited, aged 60+, totaling 186 eligible participants. The inclusion criteria were as follows: (a) Preserved capacity to communicate with others. (b) absence of medical diagnosis of neurodegenerative diseases or any other disease that may hinder exercise performance; (c) capacity to perform exercise, according to each LTCI physician; (d) no record of severe cardiopathy; (e) absence of acute musculoskeletal injuries that may hamper exercise performance; and (f) no severe sequels of cerebrovascular accident. Participant demographic data was analyzed, and the Brazilian version of the Mini-Mental State Examination (MMSE) was applied (11). NO MMSE cut-off point was applied to exclude participants.

Frailty syndrome was assessed by five objectively measured components, namely non-intentional weight loss, self-reported exhaustion, low physical activity levels, slow walking, and grip strength, leading to classifications of frail, pre-frail, and robust (12).

### Participant Randomization and Allocation

Randomization was applied with the division of parallel groups according to similar age. An independent researcher performed the procedure and used an Excel sequence of random numbers, with no participant identification. The codes of each older person's group allocation were sent to the data collection chief researcher to determine the intervention groups.

### Interventions

Interventions were performed twice a week, totaling 16 sessions across approximately 2 months. Each session lasted 30–45 min. Participants were randomly allocated to the exergames and active control groups (EG and ACG, respectively). The EG performed exercises with 2D exergames, while the ACG used the same movements as EG without virtual reality. Both intervention programs have been previously published and detailed by our laboratory (13, 14). The frequency, number of sessions, and duration were the same for both groups.

### Outcomes

#### Spatial Navigation Assessment

The Floor Maze Test - FMT (15) was used to assess spatial navigation. This test evaluates planning, allocentric spatial navigation, and episodic memory. FMT consists of a bidimensional white maze drawn in a dark blue carpet (6 m^2^). Participants must find the exit of the maze as quickly as possible. Course planning time (PT), immediate course performance maze time (IMT), and maze course repetition time without previous planning (Delayed Maze Time - DMT) were assessed. To perform the DMT, the individual remained in a room for 10 min without visual contact with the maze. IMT and DMT trajectory errors were recorded.

### Statistical Procedures

The Shapiro-Wilk and Levene tests were used to verify data normality and homoscedasticity, respectively. Descriptive analyses (mean, standard-deviation, median, and 95% confidence interval) were used for sample characterization and result presentation. Differences between post- and pre-intervention data (Δ) were estimated using the spatial navigation results. The independent *T* test and Kruskal-Wallis were used, when suitable, to compare independent group data (EG Δ vs. ACG Δ). Analyses considered α ≤ 0.05 and β = 0.20 parameters and were performed using the Statistics Package for Social Sciences (SPSS) 24.0. As the multiple comparisons inflate the alpha value, we did not perform paired analyses (within groups), to avoid Type I Errors [See (16)].

### Ethical Procedure

This research was approved by the Research Ethics Committee of the State University of Montes Claros under n. 2.398.863/2017. Brazilian Ministry of Health rules were met, according to law n. 466/2012.

## Results

**Figure 1:**
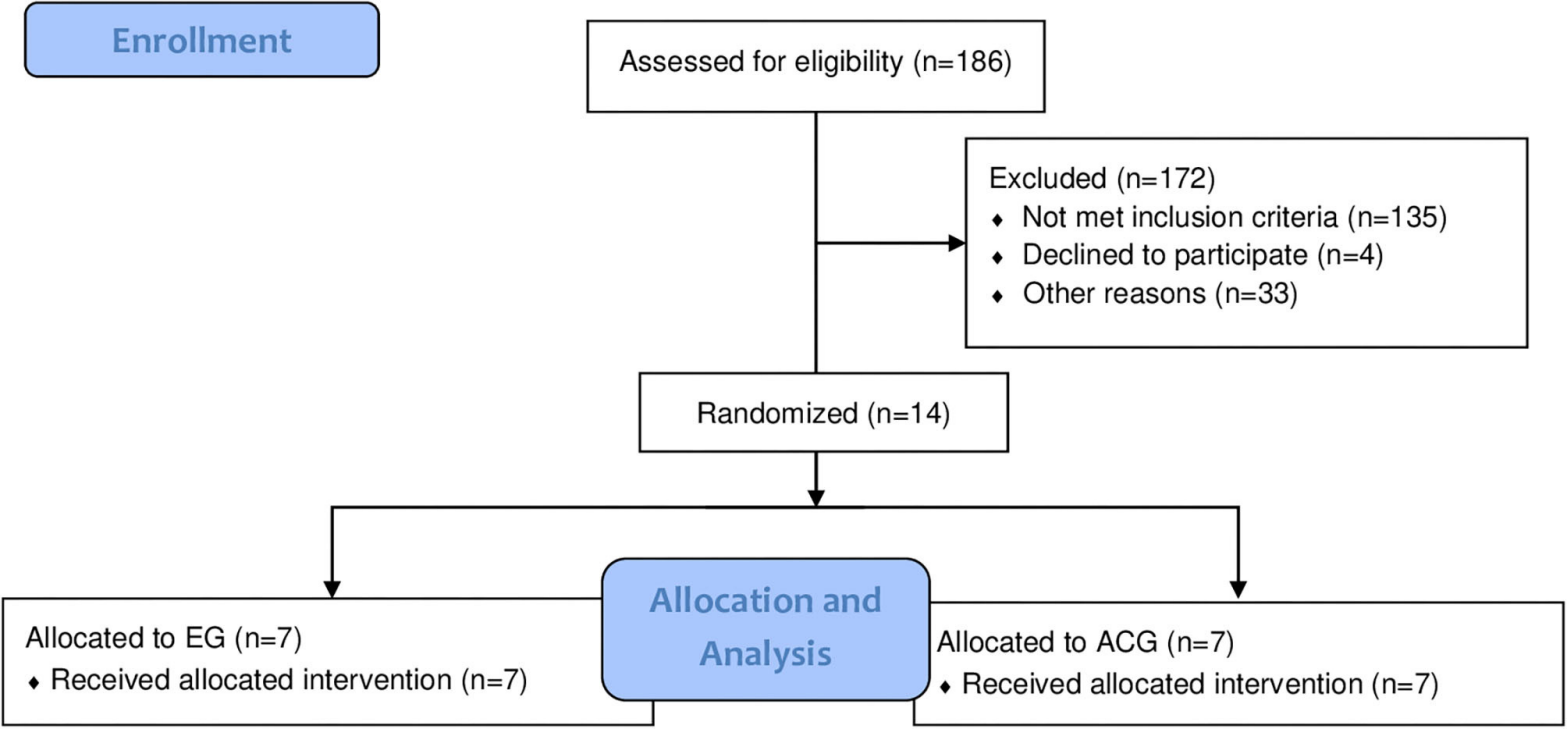
Flowchart indicating the recruitment procedure.

From the 186 eligible older persons, 14 were selected for the interventions, as all remained in the study until the end (Figure 1). Groups presented homogeneity regarding age, body weight, height, and global cognition (Table 1). All older persons exhibited frailty criteria, with 12 classified as frailty and two as prefrail.

**Table 1:** Demographic, global cognition, and spatial navigation data.

**Variable**	**EG (*****n*** **=** **7)**		**ACG (*****n*** **=** **7)**		**t/U**	**df**	***p*-value**
	**Mean**	**SD**	**Mean**	**SD**			
Age_years_	81.28	9.74	85.14	6.98	0.85^*^	12	0.41
Weight_kg_	57.58	17.68	68.35	15.19	1.22^*^	12	0.24
Height_m_	1.55	1.10	1.53	0.09	0.38^*^	12	0.70
MMSE_score_	20.8	6.6	24.0	4.8	−1.0^*^	12	0.33
IMT_seconds_	384	385	146	104	14.00^#^	–	0.20
DMT_seconds_	354	329	101	59	18.00^#^	–	0.45
	**Frequency**^**EG**^		**Frequency**^**ACG**^		**χ^2^**	**df**	***p*****-value**
Gender_M/F_	1/6		2/5		2.91	1	0.08

*Mini-Mental State Examination (MMSE) Status Examination. EG, exergame group; ACG, active control group; M, male; F, female*.

* *Independent t Test*;

# *Mann-Whitney U Test*.

**Figure 2:**
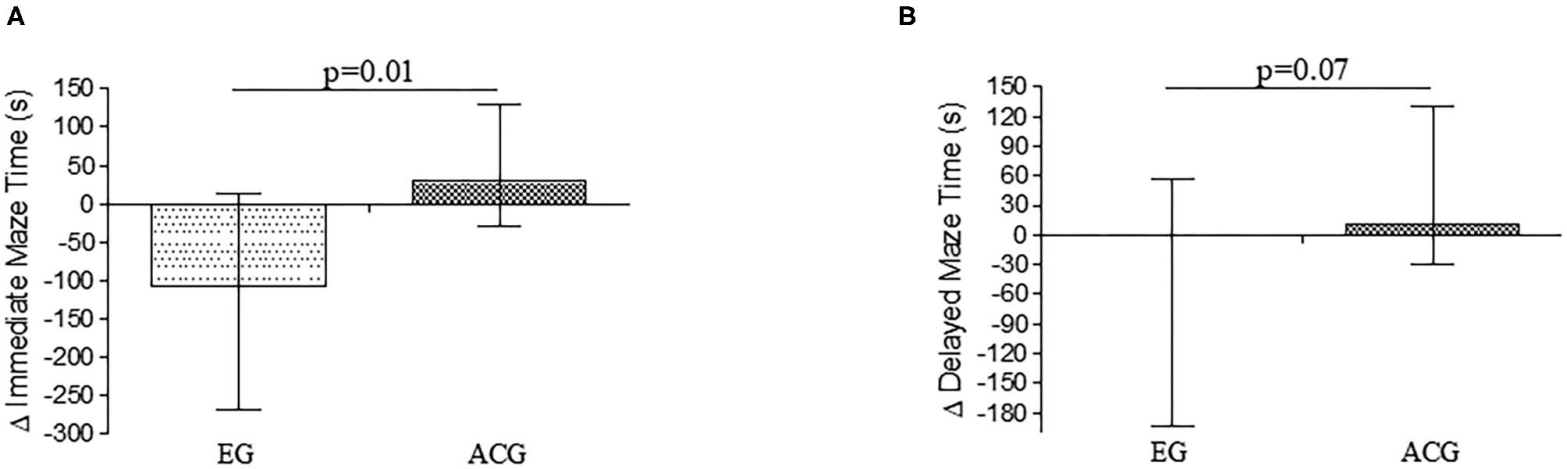
**(A)** Δ time to finish the immediate Maze Test. **(B)** Δ time to finish the Delayed Maze Test. EG: 2D Exergame Group. ACG, Active Control Group. EG IMT_baseline_: 388 ± 385 s; IMT_post−intervention_: 165 ± 147 s; EG DMT_baseline_: 354 ± 329 s; DMT_post−intervention_: 117 ± 129 s; ACG IMT_baseline_: 146 ± 104 s; IMT_post−intervention_: 200 ± 170 s; DMT_baseline_: 101 ± 59 s; DMT_post−intervention_: 150 ± 117 s.

Spatial navigation improved in EG participants compared to ACG individuals. A significant reduction in IMT performance time was observed between groups (Figure 2A), while the time to perform DMT was significantly different at the significance threshold (Figure 2B).

## Discussion

This study indicates that exercise with 2D virtual reality reduces immediate FMT performance time and improves spatial navigation in institutionalized non-robust older persons. These are promising findings regarding spatial navigation decline and speed reduction, which is a predictor of cognitive decline due to dementia disorders (6).

Spatial navigation is considered among the scientific community as a strong predictor of both cognitive impairment and Alzheimer's disease (6, 17, 18). Immediate FMT performance time is associated with executive functions and involves, mainly, planning, mental flexibility, and processing speed (14), functions which might be harmed in dementia cases and are paramount to maintaining independence and postponing institutionalization (4, 17, 19).

The main physical exercise benefits on cognition are associated with neuroplasticity, spatial learning, memory, and executive control (20–23). These benefits justify the improvement of spatial navigation ability found in the present study. Moreover, exercise with virtual reality distinguishes itself as a more attractive strategy for physical exercise practices in older individuals (24).

Other studies corroborating our findings are available, highlighting that the use of training with virtual reality improves the cognitive capacity of institutionalized older persons (10, 12, 13, 24–26). Interactions with virtual environments increase the activation of the frontoparietal cortex network, which contributes to explaining the findings reported herein, as this region is directly connected to spatial navigation (27). Furthermore, a lack of investigation on longitudinal intervention strategies on humans focusing on spatial navigation, as the one conducted in this study, is noted, as most assessments consider only acute effects.

Some studies conducted with animals have highlighted that aerobic and resistance physical exercise may result in positive interferences on brain structures associated with spatial navigation, such as the hippocampus, through the enhancement of trophic factor secretion, brain derived neurotrophic factor (BDNF), and growth factor similar to insulin type 1 (IGF-1), which promote neurogenesis (22, 28).

The most important and main limitation of this study is the sample size. Although *p*-value and statistical power were statistically significant, this finding should be interpreted with caution, as a replication of the experiment applied herein is required.

## Conclusions

Exercise with 2D virtual reality improves the spatial navigation ability of institutionalized, non-robust, older persons. The results reported herein may aid in developing strategies to improve spatial navigation capacity in institutionalized older persons and, therefore, prevent or slow down the development of prodromal dementia in this population.

## Data Availability Statement

The raw data supporting the conclusions of this article will be made available by the authors, without undue reservation.

## Ethics Statement

The studies involving human participants were reviewed and approved by Comitê de Ética em Pesquisa da Universidade Estadual de Montes Claros. Protocol number: no. 2.398.863/2017. The patients/participants provided their written informed consent to participate in this study.

## Author Contributions

LO wrote the manuscript and collected the data. EE wrote the manuscript. MA participated in the data collection. LC partially wrote, revised the content, and translated the manuscript. DF participated in the data collection. AdP revised the manuscript content and performed the analyses. KE revised the manuscript, the analyses, and the English language. RM-J established the study objective, participated in the data collection, wrote the manuscript, and analyzed the data. ON revised the manuscript, language and analyses. All authors contributed to the article and approved the submitted version.

## Conflict of Interest

The authors declare that the research was conducted in the absence of any commercial or financial relationships that could be construed as a potential conflict of interest.
